# Post-marketing surveillance of the safety and effectiveness of naldemedine in the management of opioid-induced constipation in patients with cancer pain in Japan

**DOI:** 10.1007/s00520-022-06807-y

**Published:** 2022-01-19

**Authors:** Keiko Takata, Masami Nakazawa, Keiichi Honda, Sayo Hashimoto

**Affiliations:** 1 Postmarketing Surveillance & Pharmacoepidemiology Department, Shionogi Pharmacovigilance Center Co., Ltd, Osaka, Japan; 2 Pharmacovigilance Department, Shionogi & Co., Ltd, 1-8, Doshomachi 3-chome, Chuo-ku, Osaka, 541-0045 Japan

**Keywords:** Naldemedine, Cancer, Opioid-induced constipation (OIC), Post-marketing surveillance, Safety, Japan

## Abstract

**Purpose:**

This prospective post-marketing surveillance (PMS) was designed to collect data on the safety and effectiveness of naldemedine in routine clinical practice in patients with opioid-induced constipation (OIC) and cancer pain in Japan and explore the characteristics of patients prone to diarrhea.

**Methods:**

The enrolled patients received naldemedine (0.2 mg, once a day) orally for up to 12 weeks. In the safety analysis, adverse drug reactions (ADRs), including diarrhea as a special interest, were assessed. Effectiveness was evaluated, especially regarding the frequency and condition of bowel movement.

**Results:**

In the safety analysis set (*n* = 1177), 145 ADRs occurred in 133 (11.30%) patients, and diarrhea was the most frequent event (*n* = 107, 9.09%). Most cases of diarrhea were non-serious (98.1%). Most ADRs were non-serious (93.8%), and they resolved within 2 weeks (75.9%). No patient characteristics influenced the risk of diarrhea development or aggravation. Both the frequency (75.0% and 83.2%) and condition of bowel movement (80.0% and 88.0%) were improved at 2 and 12 weeks, respectively in the effectiveness analysis set (*n* = 953). Frequency and condition of bowel movement were also improved in patients excluded (e.g., Eastern Cooperative Oncology Group performance status was ≥ 3) or with very small numbers (e.g., received weak opioid) in the clinical trials.

**Conclusions:**

This PMS indicates that naldemedine is well tolerated and effective in patients of various backgrounds in routine clinical practice who have OIC and cancer pain.

**Trial registration:**

UMIN000042851.

**Supplementary Information:**

The online version contains supplementary material available at 10.1007/s00520-022-06807-y.

## Introduction

Opioid-induced bowel dysfunction (OIBD) is a common side effect associated with the use of opioids, which are frequently prescribed for pain relief in patients with moderate-to-severe chronic or cancer pain [1]. Among OIBD symptoms, opioid-induced constipation (OIC) is the most common, being reported to occur in 51–87% and 41–57% of opioid-treated patients with cancer pain and chronic non-cancer pain, respectively [2]. Although laxatives have often been used to treat OIC [2], they are associated with various adverse effects and do not address the predominant underlying cause of the constipation [3]. A consensus statement from a European panel of experts recommended peripherally acting μ-opioid receptor antagonists (PAMORAs) or alternative opioid receptor antagonists (with or without the addition of laxatives, secretagogues, or prokinetics) for the treatment of OIC [2].

Naldemedine [Symproic® (Japan; USA); Rizmoic® (UK and Europe)] is an orally active PAMORA. Because naldemedine blocks μ-opioid receptors without readily crossing the blood-brain barrier, it is expected to treat OIC without reducing the analgesic effects of opioids, unlike centrally acting opioid receptor antagonists [4–6]). Naldemedine has demonstrated efficacy and safety in seven clinical studies in adults with OIC and cancer or chronic non-cancer pain [7–11].

However, these clinical trials were conducted under restricted conditions (e.g., patients who had never taken laxatives for the treatment of OIC and patients with an Eastern Cooperative Oncology Group performance status (ECOG-PS) of ≥ 3 were excluded, and only a small number of weak opioid-treated patients were included). In comparison to clinical trials, patients in routine clinical practice have more complications or use a wide variety of medications concomitantly. Therefore, it is extremely important to reconfirm the safety and effectiveness of naldemedine in the real-world setting. In addition, while diarrhea is the most frequently reported adverse drug reaction (ADR) of naldemedine [8, 12]), to the best of our knowledge, no patient characteristic associated with the development of diarrhea has been identified.

We conducted this post-marketing surveillance (PMS) to evaluate the safety and effectiveness of naldemedine in routine clinical practice, and to explore the characteristics of patients who are prone to develop or experience exacerbation of diarrhea.

## Methods

### Study design and population

This prospective PMS was conducted at 269 hospitals and clinics in Japan between January 2018 and June 2020 and registered with University Hospital Medical Information Network (UMIN000042851). The target number of patients was 1200. Patients with OIC and cancer pain who had never been treated with naldemedine were enrolled. The upper limit on the number of enrolments per hospital/clinic was set at 50 patients to prevent standout registrations from a particular facility. The patients were registered centrally during the registration period, and an electronic data capture (EDC) system was used to record all data for the surveillance. We collected the enrolled patients’ information, including initials, date of birth or age, sex, and date of administration of naldemedine, at the time of registration. The physician in charge of the surveillance entered and submitted the aforementioned information of enrolled patients via the EDC system. Registration was conducted within 7 days of the initiation of naldemedine treatment. The patients were administered naldemedine 0.2 mg once a day for up to 12 weeks.

### Surveillance data

We collected data at 2, 4, 8, and 12 weeks after the initiation of naldemedine treatment or the points of discontinuation/completion of naldemedine treatment. All data were entered into the EDC system by physicians. In addition to the background/demographic details of each patient, the following data were collected:

- complications (hepatic, renal, or other) and history of GI disorders
- data relating to naldemedine treatment including dose, treatment period, and reasons for treatment discontinuation/completion
- route of administration, dose, and treatment period of opioids that the patients received from 2 weeks prior to starting naldemedine treatment to the end of naldemedine treatment
- route of administration, dose, and treatment period of laxatives that the patients received from 2 weeks prior to starting naldemedine treatment to the end of naldemedine treatment
- route of administration, dose, and treatment period of non-opioid concomitant drugs that the patients received

Adverse events (AEs) and ADRs that developed after the initiation of naldemedine treatment were recorded. ADRs were defined as AEs for which their causality (as determined by the reporting physician or sponsor’s physician) could not be excluded. AEs were classified by System Organ Class and Preferred Term using Medical Dictionary for Regulatory Activities version 23.0. The deterioration of constipation and progression of malignancy (including metastases and any associated symptoms) were not regarded as AEs. However, death caused by the progression of cancer (including metastases) was regarded as a serious AE, and its causality with naldemedine treatment was determined by both the reporting and sponsor’s physician.

The effectiveness of naldemedine was qualitatively evaluated by surveillance physicians based on medical interviews with patients. We assessed the effectiveness of treatment based on improvement in the frequency of bowel movement (improved, unchanged, or worsened) and condition of bowel movement (improved, slightly improved, unchanged, slightly worsened, or worsened) including stool hardness, straining, and sensation of incomplete evacuation at each evaluation point compared with the findings prior to naldemedine treatment. We defined “improved” and “slightly improved” as improved bowel movement. We defined patients who were administered weak opioids at the time of naldemedine treatment initiation as the weak opioid treatment group, and patients who were administered strong opioids at the time of naldemedine treatment initiation comprised the strong opioid treatment group. The classification of strong and weak opioids is presented in Supplementary Table 1.

### Sample size

The cumulative incidence of diarrhea as a serious ADR was 0.9% (2/224 patients) in phase II and III clinical studies of naldemedine [8, 12]). Based on these data, 1200 patients were set as a target number of cases necessary to detect a significant twofold increase in the proportion of cumulative incidence to 1.8% during this PMS (two-tailed test at a significance level of 5% and a statistical power of 80%).

### Statistical analyses

The cumulative incidence of ADRs was calculated in the safety population. Factors that might affect the cumulative ADR incidence of naldemedine treatment were assessed using the chi-squared test to evaluate independence between categorical variables, and the Cochran-Armitage test was used to evaluate the trend associated with these variables. A significance level of 0.05 was used for all tests. Cases in the “unknown” category were excluded from statistical testing. For effectiveness outcomes, the proportion of patients with improvement and 95% confidence interval (CI) were calculated using the Clopper-Pearson method. The cumulative incidence of AEs and ADRs was rounded to the second decimal place, and other proportions were rounded to the first decimal place. The statistical software used was SAS 9.2 and later (SAS Institute Inc., Cary, NC, USA).

## Results

**Fig. 1:**
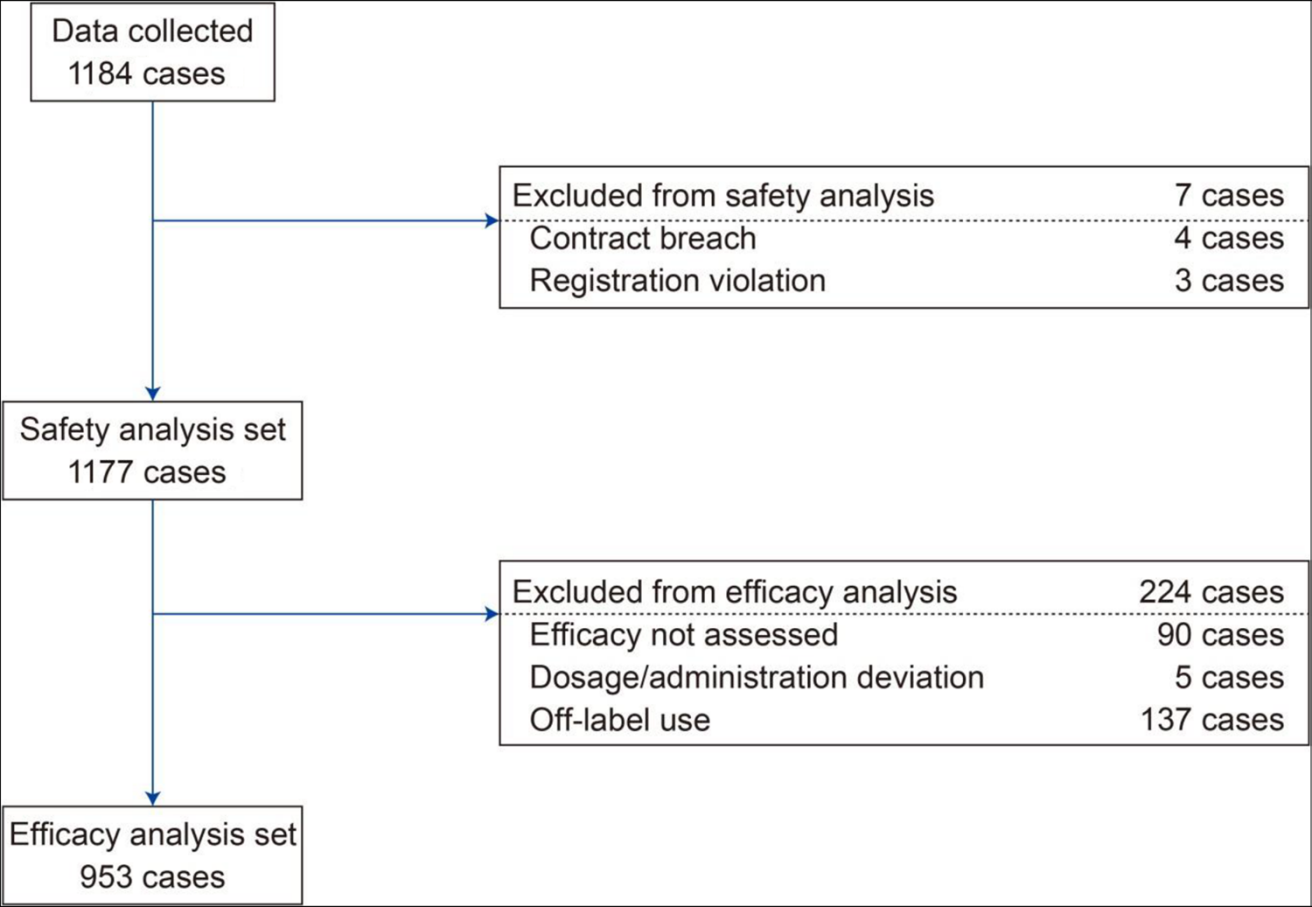
Patient disposition

In total, 1202 patients were registered for the surveillance, and 1184 case report forms were collected. Of these, seven patients were excluded from the safety analyses because of breach of contract (*n* = 4) and registration violation (*n* = 3), and 224 were excluded from the effectiveness analyses, mainly because of off-label drug usage (*n* = 137), non-assessment of effectiveness (*n* = 90), and dosage and administration deviation (*n* = 5). Patients could be excluded for more than one reason (Fig. 1).

Patient demographics, baseline characteristic, and treatment factors in the safety (*n* = 1177) and effectiveness analysis sets (*n* = 953) are presented in Table 1. In the safety analysis set, patients had a mean (SD) age of 69.0 (12.8) years old. In total, 70.1% were ≥ 65 years old, 57.1% were male, and most patients had an ECOG-PS of 1 (30.2%), 2 (27.2%), or ≥ 3 (32.4%). Nearly two-thirds of patients (64.1%) had complications. The proportions of patients with hepatic or renal function abnormalities were 9.7% and 6.3%, respectively, and 18.7% of patients had a history of GI disease. Cancer and associated metastases were not included in the complications. Death caused by the progression of cancer (including metastases) was regarded as a serious AE; however, the causality with naldemedine treatment was denied by both reporting and sponsor’s physicians. Although one patient in the safety analysis set was administered a naldemedine dose of less than 0.2 mg, all other patients in the safety analysis set received a dose of 0.2 mg daily. Nearly one-third of patients (32.5%; *n* = 383) continued naldemedine treatment throughout the 12-week surveillance. The most common reasons for treatment discontinuation in the safety analysis set were difficulty taking naldemedine because of aggravated cancer conditions (43.3%; *n* = 344), followed by cancer death (20.0%; *n* = 159), onset of AEs (7.6%; *n* = 60), discontinuation of opioids (6.9%; *n* = 55), improvement in conditions (5.5%; *n* = 44), insufficient effect (2.4%; *n* = 19), and others (14.2%; *n* = 113). In 53.9% of patients, the time from starting opioids to initiating naldemedine treatment was ≥ 14 days. The majority of patients (87.7%) received strong opioids, and 7.7% received weak opioids. Meanwhile, 72.6% (*n* = 854) of patients took laxatives before naldemedine treatment, and 776 (90.9%) of these 854 patients continued to take laxatives after starting naldemedine treatment. Most patients used concomitant laxatives (80.7%; *n* = 950), mainly osmotic/saline laxatives (63.2%; *n* = 744) and/or stimulant/colorectal-stimulant laxatives (33.6%; *n* = 396).

**Table 1:** Patient demographics, baseline characteristics and treatment factors

Parameter	Safety analysis set (*n* = 1177)	Effectiveness analysis set (*n* = 953)
Mean (SD) age, years	69.0 (12.8)	68.9 (12.9)
Sex male/female, n (%)	672 (57.1)/505 (42.9)	543 (57.0)/410 (43.0)
Eastern Cooperative Oncology Group performance status, n (%)		
0	119 (10.1)	96 (10.1)
1	356 (30.2)	283 (29.7)
2	320 (27.2)	266 (27.9)
3	298 (25.3)	247 (25.9)
4	83 (7.1)	60 (6.3)
Unknown	1 (0.1)	1 (0.1)
Primary focus, n (%)		
Lung cancer	199 (16.9)	157 (16.5)
Pancreatic cancer	149 (12.7)	119 (12.5)
Breast cancer	90 (7.6)	70 (7.3)
Gastric cancer	79 (6.7)	67 (7.0)
Colon cancer	53 (4.5)	46 (4.8)
Others	617 (52.4)	502 (52.7)
Hepatic function abnormalities, n (%)	114 (9.7)	91 (9.5)
Renal function abnormalities, n (%)	74 (6.3)	56 (5.9)
Complications (complications excluding cancer and its metastasis), n (%)	755 (64.1)	622 (65.3)
History of GI disease, n (%)	220 (18.7)	182 (19.1)
Treatment factors		
Duration of naldemedine treatment (days), Median (Q1, Q3)	42.0 (17.0, 85.0)	47.0 (21.0, 85.0)
Duration of naldemedine treatment, n (%)		
< 2 weeks	220 (18.7)	139 (14.6)
2– < 4 weeks	215 (18.3)	183 (19.2)
4– < 6 weeks	142 (12.1)	124 (13.0)
6– < 8 weeks	81 (6.9)	62 (6.5)
8– < 10 weeks	83 (7.1)	73 (7.7)
10– < 12 weeks	53 (4.5)	44 (4.6)
≥ 12 weeks	383 (32.5)	328 (34.4)
Time from opioid administration to starting naldemedine, n (%)		
1–2 days	219 (18.6)	95 (10.0)
3–4 days	105 (8.9)	95 (10.0)
5–6 days	69 (5.9)	63 (6.6)
7–13 days	134 (11.4)	117 (12.3)
≥ 14 days	634 (53.9)	577 (60.5)
Unknown	16 (1.4)	6 (0.6)
Types of opioid analgesics when naldemedine was started, n (%)		
Weak	91 (7.7)	77 (8.1)
Strong	1032 (87.7)	833 (87.4)
Weak + strong	47 (4.0)	42 (4.4)
Unknown	7 (0.6)	1 (0.1)
Opioid exposure within 2 weeks before the start of naldemedine (morphine equivalent), Median (Q1, Q3)	265.0 (90.0, 630.0)	262.5 (90.0, 630.0)
Previous use of laxatives (including prophylactic*), n (%)	854 (72.6)	747 (78.4)
Concomitant laxatives, n (%)	950 (80.7)	811 (85.1)
Osmotic laxatives/saline laxatives	744 (63.2)	647 (67.9)
Stimulant laxatives/Colorectal stimulant laxatives	396 (33.6)	344 (36.1)
Chloride channel activators	82 (7.0)	75 (7.9)
Osmotic laxatives/Carbohydrate laxatives	31 (2.6)	29 (3.0)
Enemas	30 (2.5)	27 (2.8)
Guanylate cyclase C receptor agonist	28 (2.4)	27 (2.8)
Ileal bile acid transporter inhibitors	19 (1.6)	16 (1.7)
Osmotic laxatives/polyethylene glycol	4 (0.3)	4 (0.4)
Others	69 (5.9)	56 (5.9)
Concomitant drugs other than opioids or laxatives, n (%)	996 (84.6)	812 (85.2)

Abbreviations: d, days; w, weeks; n, number of patients; Q, quartile; SD, standard deviation;

^*^prescription prior to initiation of opioids

### Safety

**Fig. 2:**
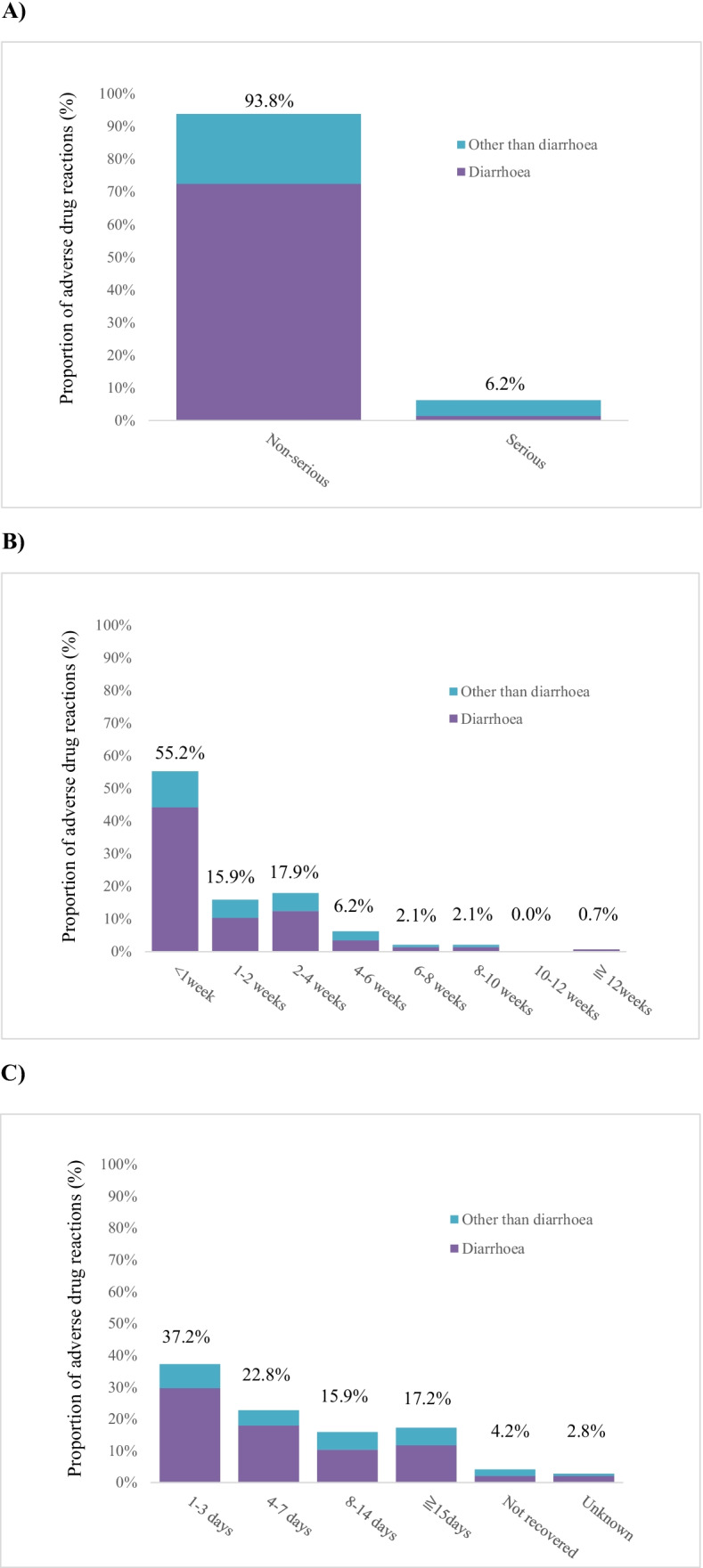
A) Proportion of serious and non-serious adverse drug reactions (ADRs). B) Time to onset of ADR after the start of naldemedine treatment. C) Time to recovered and recovering after onset of ADR (day confirmed by the physician)

AEs were observed in 756 (64.23%) patients. The majority of AEs were symptoms associated with cancer progression. The causal relationship with naldemedine treatment was denied by both reporting and the sponsor’s physician. In total, 145 ADRs occurred in 133 (11.30%) patients, with most cases (*n* = 121; 10.28%) being GI disorders (Table 2). Of the reported 145 events, 136 (93.8%) events were non-serious, more than half (55.2%) of the events developed within the first week of naldemedine treatment, and most events resolved within 1 (60.0%) or 2 weeks (75.9%, Fig. 2).

**Table 2:** Number of cases with adverse drug reactions in the safety population (*n* = 1,177)

**Cases with Adverse Drug Reactions, total**		**n (%)** 133 (11.30)
System Organ Class	Preferred Term	
Infections and infestations		1 (0.08)
	Pneumonia	1 (0.08)
Metabolism and nutrition disorders		4 (0.34)
	Dehydration	1 (0.08)
	Hyperkalemia	1 (0.08)
	Hypokalemia	1 (0.08)
	Decreased appetite	1 (0.08)
Psychiatric disorders		4 (0.34)
	Delirium	2 (0.17)
	Insomnia	2 (0.17)
Gastrointestinal disorders		121 (10.28)
	Abdominal discomfort	1 (0.08)
	Abdominal pain	8 (0.68)
	Abdominal pain lower	1 (0.08)
	Constipation	1 (0.08)
	Diarrhea	107 (9.09)
	Gastrointestinal pain	1 (0.08)
	Nausea	3 (0.25)
	Vomiting	1 (0.08)
	Large intestinal hemorrhage	1 (0.08)
	Feces soft	3 (0.25)
	Anal incontinence	1 (0.08)
Hepatobiliary disorders		1 (0.08)
	Hepatic function abnormal	1 (0.08)
Skin and subcutaneous tissue disorders		3 (0.25)
	Drug eruption	1 (0.08)
	Hyperhidrosis	1 (0.08)
	Rash	1 (0.08)
General disorders and administration site conditions		2 (0.17)
	Inadequate analgesia	1 (0.08)
	Edema peripheral	1 (0.08)
Investigations		1 (0.08)
	Alanine aminotransferase increased	1 (0.08)
	Aspartate aminotransferase increased	1 (0.08)

There were nine serious ADRs in seven patients, namely two cases of diarrhea, two cases of delirium, and one case each of pneumonia, dehydration, hyperkalemia, vomiting, and large intestinal hemorrhage. Seven serious ADRs in five patients were recovered or recovering. A patient with hyperkalemia with an unknown outcome was transferred to another hospital, and the patient with large intestinal hemorrhage died because of a tumor. No evidence of GI perforation, an important potential risk in the risk management plan (RMP), was found in this patient.

Diarrhea, an important identified risk in the RMP, was the most frequently observed ADR (*n* = 107, 9.09%, Table 2). Most cases (98.1%) of diarrhea were non-serious; however, two cases were assessed as serious. One of the two serious cases occurred in a patient with an extended hospital stay because of exacerbation of diarrhea that occurred prior to the initiation of naldemedine treatment. The patient recovered following the discontinuation of naldemedine and by the treatment of loperamide 6 days after the exacerbation of diarrhea. The other patient was hospitalized to treat dehydration, and the condition recovered following the discontinuation of naldemedine treatment with an intravenous drip infusion. Of 107 patients with diarrhea, 77 had experienced recovery, 24 were recovering, and the outcome was not confirmed in six patients, who were transferred to other hospitals. The patient background and treatment factors in the incidence cases of diarrhea are presented in Table 3. Statistically significant differences of the incidence of diarrhea in patient background characteristics were observed for “complications (excluding cancer)” (*p* = 0.0163), “duration of naldemedine treatment” (*p* = 0.0040), and “concomitant drugs other than opioids or laxatives” (*p* = 0.0079, Table 3).

**Table 3:** Incidence of diarrhea as adverse drug reactions by patient background and treatment factors

Background and treatment factors	Patients (n/total)	Proportion (%)	95% CI	*P* value
All cases	107/1177	9.09	7.510 to 10.880	–
**Patient background**				
Age group				
15–64 years	35/352	9.94	7.024 to 13.557	0.5065
≥ 65 years	72/825	8.73	6.891 to 10.864	
Sex				
Male	57/672	8.48	6.487 to 10.850	0.4020
Female	50/505	9.90	7.438 to 12.845	
Eastern Cooperative Oncology Group performance status				
0	10/119	8.40	4.103 to 14.911	0.5271^1^
1	38/356	10.67	7.665 to 14.356	
2	30/320	9.38	6.415 to 13.113	
3	25/298	8.39	5.503 to 12.135	
4	4/83	4.82	1.329 to 11.882	
Unknown	0/1	0.00		
Hepatic function abnormalities present	11/114	9.65	4.916 to 16.609	0.8273
Hepatic function abnormalities absent	96/1063	9.03	7.376 to 10.917	
Renal impairment present	9/74	12.16	5.715 to 21.836	0.3424
Renal impairment absent	98/1103	8.88	7.272 to 10.721	
Complications (except cancer) present	80/755	10.60	8.492 to 13.014	0.0163
Complications (except cancer) absent	27/422	6.40	4.258 to 9.173	
History of gastrointestinal disease present	25/220	11.36	7.490 to 16.317	0.1930
History of gastrointestinal disease absent	81/946	8.56	6.858 to 10.530	
**Treatment factors**				
Duration of naldemedine treatment				
< 2 weeks	35/220	15.91	11.338 to 21.425	0.0040^2^
2– < 4 weeks	20/215	9.30	5.775 to 14.001	
4– < 6 weeks	8/142	5.63	2.463 to 10.799	
6– < 8 weeks	8/81	9.88	4.361 to 18.536	
8– < 10 weeks	4/83	4.82	1.329 to 11.882	
10– < 12 weeks	6/53	11.32	4.270 to 23.029	
≥ 12 weeks	26/383	6.79	4.482 to 9.789	
Time from opioid administration to starting naldemedine treatment				
1–2 days	13/219	5.94	3.198 to 9.937	0.2471
3–4 days	14/105	13.33	7.485 to 21.358	
5–6 days	7/69	10.14	4.177 to 19.792	
7–13 days	11/134	8.21	4.169 to 14.213	
≥ 14 days	62/634	9.78	7.580 to 12.361	
Unknown	0/16	0.00	-	
Opioid analgesics used when naldemedine was started				
Weak	12/91	13.19	7.004 to 21.902	0.2384
Strong	89/1032	8.62	6.983 to 10.506	
Weak + strong	6/47	12.77	4.832 to 25.741	
Unknown	0/7	0.00	-	
Previous use of laxatives (including prophylactic): yes	86/854	10.07	8.134 to 12.287	0.0574
Previous use of laxatives (including prophylactic): no	21/323	6.50	4.069 to 9.767	
Concomitant laxatives: yes	91/950	9.58	7.782 to 11.630	0.2335
Concomitant laxatives: no	16/227	7.05	4.082 to 11.194	
Concomitant drugs other than opioids or laxatives: yes	100/996	10.04	8.244 to 12.077	0.0079
Concomitant drugs other than opioids or laxatives: no	7/181	3.87	1.569 to 7.806	

^1^P_trend_ = 0.2287; ^2^ P_trend_ 0.0036; ^3^ P_trend_ = 0.3189. All p_trend_ results were calculated using the Cochran-Armitage test

There were no ADRs concerning opioid withdrawal syndrome, GI perforation, and cardiovascular events, which are important potential risks in the RMP. One patient required an increase in the opioid dose after the administration of naldemedine. A reduced analgesic effect of opioids following naldemedine treatment, which is also an important potential risk in the RMP, was suspected, but underlying disease or complications were also possible factors to have caused the event.

Concerning the patients who experienced ADRs, 44.1% of events did not affect their dosing regimen of naldemedine.

### Effectiveness

**Fig. 3:**
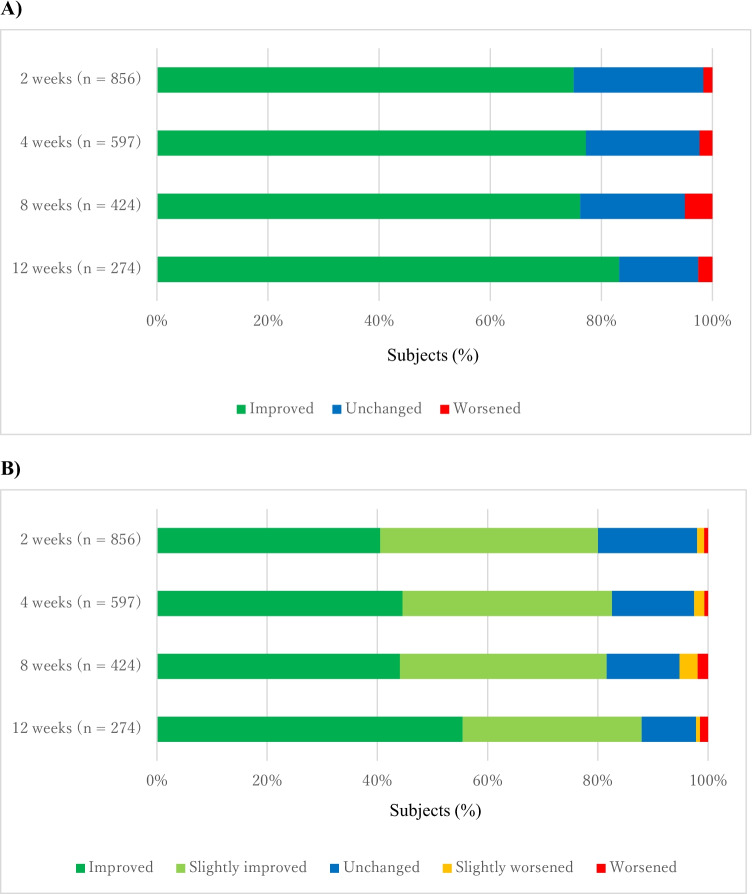
A) Improvement in frequency of bowel movement. B) Improvement in condition of bowel movement

In the effectiveness analysis set (*n* = 953), improvement of the frequency of bowel movement was observed in week 2 [75.0% (95% CI = 71.96–77.87)], and the improvement was maintained through week 12 [83.2% (95% CI = 78.25–87.44)] (Fig. 3A and Supplemental Table 3A). Similarly, an improvement of the condition of bowel movement was observed in week 2 [80.0% (95% CI = 77.19–82.65)] and maintained through week 12 [88.0% (95% CI = 83.50–91.56)] (Fig. 3B and Supplemental Table 3B).

We searched for patient’s backgrounds and therapeutic factors that affect the frequency and condition of bowel movements (Supplementary Table 2), including populations who had not been examined much in the previous clinical trials [8, 11]). Improvements in frequency and condition of bowel movements at week 12 were observed in patients with ECOG-PS = 3 (88.2% (30/34) and 88.2 (30/34), respectively) or ECOG-PS = 4 (81.8% (9/11) and 81.8% (9/11), respectively). In addition, 77.8% (21/27) and 88.9% (24/27) of patients who received weak opioids experienced improvement of the condition of bowel movement at 2 and 12 weeks, respectively, compared with 84.5% (197/233) and 88.4% (206/233), respectively, of patients who received strong opioids (Supplementary Table2). Other results are also available in Supplementary Table 2.

## Discussion

The safety and effectiveness of naldemedine 0.2 mg were assessed in 1177 and 953 patients with OIC and cancer pain in routine clinical practice, respectively. The results suggested that naldemedine treatment was well tolerated and effective in routine clinical practice. Among 1177 patients in the safety population, 133 patients experienced 145 ADRs. Nine serious ADRs were reported in seven patients. Concerning the six serious ADRs other than diarrhea and dehydration, the contribution of patients’ background conditions (cancer and opioid use) was highly suspected. No patients recovered with sequelae or had a fatal outcome.

There were two patients reported to be hospitalized due to serious diarrhea in the previous clinical trial. Both patients recovered following discontinuation of naldemedine and fluid replacement [11]. Therefore, diarrhea is listed as an important identified risk in the RMP for naldemedine and occurrence of severe diarrhea is stated in the package insert to be an ADR of special attention.

Most ADRs in this surveillance were diarrhea and other GI symptoms. Diarrhea was observed in 107 patients (9.09%). All but two events were deemed non-serious. The incidence of diarrhea was lower in patients without “complications (except cancer)” and in patients who did not receive “concomitant drugs other than opioids or laxatives” (Table 3). However, we could not find any reason to explain these observations. Patients treated with naldemedine for less than 2 weeks had a higher incidence of diarrhea (Table 3). This is likely to be because diarrhea occurred during the first weeks of naldemedine treatment, and many patients developing the ADR discontinued naldemedine treatment shortly afterwards. No factors considered to affect the development or aggravation of diarrhea were identified.

The incidence of ADRs other than diarrhea or GI symptoms was limited, and they were observed sporadically without a specific pattern. No ADRs concerning other important risks in the RMP were observed excluding one suspected case of a reduced opioid analgesic effect. However, it cannot be ruled out that pain may have increased in the patient with cancer progression. Most ADRs including diarrhea occurred in the early phase of treatment, and nearly half of the cases required no change in the dosing regimen or discontinuation of naldemedine treatment.

Compared with the results of clinical trials (85.6% for the naldemedine group in COMPOSE-4 and 81.7% in COMPOSE-5) [8, 11]), the proportion of patients who continued treatment was low in this surveillance (32.5%). However, most reasons for the discontinuation of naldemedine treatment were cancer progression (i.e., “cancer-related death” and “difficulty taking the drug due to cancer progression”). The majority of events, excluding cancer progression, which caused patients to withdraw from surveillance or clinical trials were GI symptoms. One possible reason might be the patients’ ECOG-PS, which was 0–2 in the clinical trials, compared with 0–4 in this surveillance. The percentage of patients who withdrew because of AEs was 7.6% in this surveillance, which is similar to the rates in COMPOSE-4 (9.3%) and COMPOSE-5 (9.2%) [8, 11]). Therefore, we can conclude that there was no difference in the safety profile between this surveillance and prior clinical trials.

Treatment with naldemedine improved the frequency of bowel movements in 75% of patients after 2 weeks of treatment and > 80% of patients after 12 weeks. In addition, the condition of bowel movement improved in 80% of patients after 2 weeks and in almost 90% of patients after 12 weeks. In clinical studies, the proportion of patients with spontaneous bowel movement at 2 weeks was approximately 70% [8], and a significant improvement from baseline was observed in Patient Assessment of Constipation (PAC)-Symptoms and PAC-Quality of Life throughout a 12-week evaluation [11]. Thus, the results of the current surveillance are considered equivalent to findings from previous clinical studies.

The number of patients available for effectiveness analyses was lower after 12 weeks than after 2 weeks because some patients discontinued naldemedine treatment. As mentioned previously, discontinuation was mainly attributable to aggravated cancer-related conditions or cancer-related deaths.

Although we cannot directly compare the results of the clinical trials and this surveillance, because the study design such as patient characteristics and evaluation methods for effectiveness on bowel movements differed among the studies, we confirmed that the effectiveness of naldemedine was maintained for 12 weeks after the initiation of treatment even in patients with a broader clinical background included in this surveillance. Although this surveillance included patients who were excluded from clinical trials, such as patients who had never taken laxatives for the treatment of OIC prior to naldemedine administration or patients whose ECOG-PS was 3 or 4, our results are consistent with results of clinical trials [7–13]. In this surveillance, 77 patients who had been administered weak opioids when naldemedine treatment started were enrolled, and 27 patients were assessed for effectiveness at 12 weeks of treatment. To the best of our knowledge, this is the first finding that suggests the effectiveness of naldemedine in patients taking both weak and strong opioids. Although further investigations are required to confirm the effectiveness in patients with OIC who received weak opioids, our results suggest that naldemedine is effective regardless of the receipt of strong or weak opioids in patients with OIC.

As diarrhea might be another issue affecting efficacy, there was a concern that the efficacy of naldemedine could be insufficient in patients who did not develop diarrhea. However, the concern could be denied because diarrhea occurred in only 9.1% of patients and effectiveness was confirmed in 75.0% of patients.

Some limitations potentially exist in this surveillance. First, is the absence of a control group. Second, the method used to evaluate the improvement and condition of bowel movement was subjective, which might lead to potential bias. Third, only patients reported in EDC were included in this surveillance, and there were some time points with smaller sample sizes due to treatment discontinuation from cancer progression, therefore these might lead to selection bias. Fourth, this surveillance was conducted only in Japan, and thus, the findings might have limited generalisability in terms of racial diversity. Lastly, although the analysis was conducted based on a fixed analysis plan, the data input into the EDC system and the interpretation of results were conducted by physicians at each institution, and it cannot be completely ruled out that the involvement of the pharmaceutical company sponsoring this surveillance might bias the results and discussion in this manuscript; however, this is a ubiquitous issue for all company-sponsored research.

In conclusion, our results demonstrated that naldemedine is well tolerated and effective in patients with OIC and cancer pain, even when those patients have various backgrounds, including characteristics found in routine clinical practice. Naldemedine should be used in consideration of the benefit-risk balance, paying attention to ADRs, especially diarrhea, during the early phase of the treatment.

## Supplementary Information

Below is the link to the electronic supplementary material.

Supplementary file1 (DOCX 31 KB)

## Data Availability

The data for this surveillance are not available in a public repository because Shionogi takes suitable measures to protect personal information and the sponsor’s intellectual property. The nature of the information protected will be tailored to the specific request. Researchers can request access to detailed information about Shionogi's clinical trials, including trial protocols and individual patient data, through the portal site: https://clinicalstudydatarequest.com/. Sharable information includes data about Shionogi's clinical trials conducted in patients in Japan. The information will become sharable after the medicinal products for which the trials are performed have been approved in Japan. Note that all documents will be provided in Japanese language only as they have been prepared in Japanese.
