# Supplementary material for: Post-marketing surveillance of the safety and effectiveness of naldemedine in the management of opioid-induced constipation in patients with cancer pain in Japan
Source: Support Care Cancer. 2022 Jan 19;30(5):3943–54. doi: 10.1007/s00520-022-06807-y (PMC8942924; doi:10.1007/s00520-022-06807-y)
Supplement: Supplementary file 1 — Supplementary file1 (DOCX 31 KB) [file 520_2022_6807_MOESM1_ESM.docx]

**Supplementary information**

**Supplementary Table 1 Definitions for strong and weak opioids in this surveillance**

| **Category** | **Drug name** |
| --- | --- |
| Strong opioids | Morphine |
|  | Oxycodone |
|  | Fentanyl |
|  | Methadone |
|  | Tapentadol |
|  | Hydromorphone |
|  | Pethidine |
|  | Buprenorphine |
| Weak opioids | Codeine |
|  | Dihydrocodeine |
|  | Tramadol |
|  | Opium |
|  | Pentazocine |
|  | Eptazocine |

**Supplementary Table 2 Frequency of bowel movements and Condition of bowel movement by patient background and treatment factors (12weeks)**

| **Background and treatment factors** | **Frequency of bowel movements** | | | | | **Condition of bowel movement** | | | | | |
| --- | --- | --- | --- | --- | --- | --- | --- | --- | --- | --- | --- |
|  | **Patients (n/total)** | **Proportion improved (%)** | **95%CI** | **P value** | **Patients (n/total)** | | | **Proportion improved (%)** | | **95%CI** | **P value** |
| All cases | 228/274 | 83.2 | 78.25 to 87.44 | - | 241/274 | | | 88.0 | | 83.50 to 91.56 | - |
| **Patient background** | | | | | | | | | | | |
| Age group | | | | | | | | | | | |
| 15–64 years | 82/105 | 78.1 | 68.97 to 85.58 | 0.0741 | 85/105 | | 81.0 | | 72.13 to 87.96 | | 0.0050 |
| ≥65 years | 146/169 | 86.4 | 80.28 to 91.17 |  | 156/169 | | 92.3 | | 87.21 to 95.84 | |  |
| Sex | | | | | | | | | | | |
| Male | 118/144 | 81.9 | 74.67 to 87.85 | 0.5547 | 127/144 | | 88.2 | | 81.77 to 92.97 | | 0.8985 |
| Female | 110/130 | 84.6 | 77.24 to 90.34 |  | 114/130 | | 87.7 | | 80.78 to 92.80 | |  |
| **Eastern Cooperative Oncology Group performance status score** | | | | | | | | | | | |
| 0 | 41/45 | 91.1 | 78.78 to 97.52 | 0.2375^1^ | 41/45 | | 91.1 | | 78.78 to 97.52 | | 0.6859^2^ |
| 1 | 102/123 | 82.9 | 75.09 to 89.11 |  | 110/123 | | 89.4 | | 82.60 to 94.25 | |  |
| 2 | 45/60 | 75.0 | 62.14 to 85.28 |  | 50/60 | | 83.3 | | 71.48 to 91.71 | |  |
| 3 | 30/34 | 88.2 | 72.55 to 96.70 |  | 30/34 | | 88.2 | | 72.55 to 96.70 | |  |
| 4 | 9/11 | 81.8 | 48.22 to 97.72 |  | 9/11 | | | 81.8 | | 48.22 to 97.72 |  |
| Unknown | 1/1 | 100 | - | - | 1/1 | | | 100 | | - | - |
| Hepatic function abnormalities: yes | 14/20 | 70.0 | 45.72 to 88.11 | 0.1006 | 16/20 | | | 80.0 | | 56.34 to 94.27 | 0.2562 |
| Hepatic function abnormalities: no | 214/254 | 84.3 | 79.18 to 88.50 |  | 225/254 | | | 88.6 | | 84.02 to 92.22 |  |
| Renal impairment: yes | 12/15 | 80.0 | 51.91 to 95.67 | 0.7321 | 13/15 | | | 86.7 | | 59.54 to 98.34 | 0.8746 |
| Renal impairment: no | 216/259 | 83.4 | 78.30 to 87.72 |  | 228/259 | | | 88.0 | | 83.44 to 91.72 |  |
| Complications (except cancer): yes | 140/174 | 80.5 | 73.78 to 86.07 | 0.1079 | 151/174 | | | 86.8 | | 80.83 to 91.43 | 0.4307 |
| Complications (except cancer): no | 88/100 | 88.0 | 79.98 to 93.64 |  | 90/100 | | | 90.0 | | 82.38 to 95.10 |  |
| History of gastrointestinal disease: yes | 48/54 | 88.9 | 77.37 to 95.81 | 0.2301 | 52/54 | | | 96.3 | | 87.25 to 99.55 | 0.0400 |
| History of gastrointestinal disease: no | 179/218 | 82.1 | 76.37 to 86.96 |  | 188/218 | | | 86.2 | | 80.94 to 90.52 |  |
| **Treatment factors** | | | | | | | | | | | |
| Duration of naldemedine treatment | | | | | | | | | | | |
| 10–<12 weeks | 17/25 | 68.0 | 46.50 to 85.05 | 0.0328 | 19/25 | | | 76.0 | | 54.87 to 90.64 | 0.0540 |
| ≥12 weeks | 211/249 | 84.7 | 79.66 to 88.97 |  | 222/249 | | | 89.2 | | 84.62 to 92.73 |  |
| *Time from opioid administration to starting naldemedine treatment* | | | | | | | | | | | |
| 1–2 days | 17/21 | 81.0 | 58.09 to 94.55 | 0.7783^3^ | 18/21 | | | 85.7 | | 63.66 to 96.95 | 0.6540^4^ |
| 3–4 days | 18/20 | 90.0 | 68.30 to 98.77 |  | 19/20 | | | 95.0 | | 75.13 to 99.87 |  |
| 5–6 days | 15/20 | 75.0 | 50.90 to 91.34 |  | 16/20 | | | 80.0 | | 56.34 to 94.27 |  |
| 7–13 days | 23/28 | 82.1 | 63.11 to 93.94 |  | 24/28 | | | 85.7 | | 67.33 to 95.97 |  |
| ≥14 days | 155/185 | 83.8 | 77.67 to 88.78 |  | 164/185 | | | 88.6 | | 83.17 to 92.83 |  |
| Opioid analgesics used when naldemedine was started | | | | | | | | | |  |  |
| Weak | 21/27 | 77.8 | 57.74 to 91.38 | 0.3229 | 24/27 | | | 88.9 | | 70.84 to 97.65 | 0.5401 |
| Strong | 197/233 | 84.5 | 79.26 to 88.94 |  | 206/233 | | | 88.4 | | 83.59 to 92.22 |  |
| Weak + strong | 10/14 | 71.4 | 41.90 to 91.61 |  | 11/14 | | | 78.6 | | 49.20 to 95.34 |  |
| Previous use of laxatives (including prophylactic): yes | 190/223 | 85.2 | 79.85 to 89.59 | 0.0653 | 201/223 | | | 90.1 | | 85.44 to 93.71 | 0.0205 |
| Previous use of laxatives (including prophylactic): no | 38/51 | 74.5 | 60.37 to 85.67 |  | 40/51 | | | 78.4 | | 64.68 to 88.71 |  |
| Concomitant laxatives: yes | 206/246 | 83.7 | 78.52 to 88.12 | 0.4881 | 218/246 | | | 88.6 | | 83.97 to 92.30 | 0.3185 |
| Concomitant laxatives: no | 22/28 | 78.6 | 59.05 to 91.70 |  | 23/28 | | | 82.1 | | 63.11 to 93.94 |  |
| Concomitant drag other than opioids or laxatives: yes | 200/243 | 82.3 | 76.91 to 86.89 | 0.2607 | 213/243 | | | 87.7 | | 82.85 to 91.51 | 0.6673 |
| Concomitant drag other than opioids or laxatives: no | 28/31 | 90.3 | 74.25 to 97.96 |  | 28/31 | | | 90.3 | | 74.25 to 97.96 |  |

1Ptrend=0.3928; 2Ptrend=0.2821; 3Ptrend=0.8605; 4Ptrend=0.8335. All ptrendresults were calculated using the Cochran-Armitage test

**Supplementary Table 3A：Improvement in frequency of bowel movement**

|  | **Patients (n)** | **Frequency of bowel movements** | | | **Proportion improved (95% CI)** | |
| --- | --- | --- | --- | --- | --- | --- |
|  |  | **Improved** | **Unchanged** | **Worsened** |  |  |
| 2 weeks | 856 | 642 (75.0 %) | 200 (23.4 %) | 14 (1.6 %) | 75.0% | (71.96 to 77.87) |
| 4 weeks | 597 | 461 (77.2 %) | 122 (20.4 %) | 14 (2.3 %) | 77.2% | (73.64 to 80.53) |
| 8 weeks | 424 | 323 (76.2 %) | 80 (18.9 %) | 21 (5.0 %) | 76.2% | (71.83 to 80.16) |
| 12 weeks | 274 | 228 (83.2 %) | 39 (14.2 %) | 7 (2.6 %) | 83.2% | (78.25 to 87.44) |

**Supplementary Table 3B：Improvement in** **condition of bowel movement**

|  | **Patients (n)** | **Condition of bowel movement** | | | | | **Proportion improved/**  **slightly improved**  **(95% CI)** |
| --- | --- | --- | --- | --- | --- | --- | --- |
|  |  | **Improved** | **Slightly**  **improved** | **Unchanged** | **Slightly**  **worsened** | **Worsened** |  |
| 2 weeks | 856 | 347 (40.5%) | 338 (39.5%) | 154 (18.0%) | 11 (1.3%) | 6 (0.7%) | 80.0% (77.19 to 82.65) |
| 4 weeks | 597 | 266 (44.6%) | 227 (38.0%) | 89 (14.9%) | 11 (1.8%) | 4 (0.7%) | 82.6% (79.29 to 85.54) |
| 8 weeks | 424 | 187 (44.1%) | 159 (37.5%) | 56 (13.2%) | 14 (3.3%) | 8 (1.9%) | 81.6% (77.58 to 85.18) |
| 12 weeks | 274 | 152 (55.5%) | 89 (32.5%) | 27 (9.9%) | 2 (0.7%) | 4 (1.5%) | 88.0% (83.50 to 91.56) |
